# Diagnostic Challenge of Small Bowel Neuroendocrine Tumour Mimicking Biliary Disease: A Case Report

**DOI:** 10.7759/cureus.108179

**Published:** 2026-05-03

**Authors:** Bisma Nazir, Ariba Mumtaz, Salman Zafar, Nimra Sadiq, Yasir Ihsan

**Affiliations:** 1 Respiratory Medicine, Blackpool Victoria Hospital, Blackpool, GBR; 2 Internal Medicine/Acute Medicine, Blackpool Victoria Hospital, Blackpool, GBR; 3 Internal Medicine, King Edward Medical University, Blackpool, GBR; 4 Diabetes and Endocrinology, Royal Lancaster Infirmary, Lancaster, GBR

**Keywords:** carcinoid markers, diagnostic challenge, emergency laparotomy, ileal neoplasm, multidisciplinary team management, neuroendocrine tumour, non-functional tumour, post-operative delirium, small bowel obstruction, surgical resection

## Abstract

Small bowel neuroendocrine tumours (NETs) are uncommon neoplasia that often occur with non-specific gastrointestinal symptoms, leading to considerable delays in diagnosis and treatment, and overlapping findings that often distract the clinical focus from the underlying pathology. We present the case of a 49-year-old woman who reported to us with a one-month history of epigastric pain, nausea, vomiting, and unintentional weight loss of about two stones. Initial imaging showed deranged liver markers and cholelithiasis on ultrasound without biliary enlargement, which resulted in a tentative diagnosis of cholecystitis. On the third day of admission, the patient acutely worsened with intense pain in the abdomen, distension, refractory vomiting, and hyperlactatemia. Acute small bowel obstruction was shown on urgent computed tomography, and an emergency laparotomy was undertaken, which revealed a circumferential stricturing ileal tumour with mesenteric lymphadenopathy, and surgical resection was done. Acute confusion and altered mental status complicated the postoperative course, necessitating further workup, which included metabolic screening, cerebral spinal fluid analysis, and neuroimaging, but they all came back clear, and the episode was considered postoperative delirium. While tumour markers were normal, histopathology revealed a well-differentiated Grade 1 NET with muscularis propria invasion and focal subserosa involvement. One-month follow-up CT showed no residual or recurrent disease, and a surveillance plan was laid out at a special NET multidisciplinary team meeting. The case underscores the difficulties in diagnosing small bowel NETs in cases where the incidental findings mask the initial pathology. This case highlights the significance of considering a wider range of differential diagnoses in patients presenting with unclear gastrointestinal symptoms to avoid potential delays in investigations and further treatment.

## Introduction

Neuroendocrine tumour (NET) is a heterogeneous type of neoplasm arising as a result of neuroendocrine cells that are located all over the body, with the gastrointestinal tract being the most frequent location of origin [1]. Over the past decades, the number of NETs has grown manifold, and the age-adjusted incidence of these diseases in England has been growing 3.7-fold, between 2.35 and 8.61 per 100,000 population [2]. This increasing incidence and relatively high survival rates relative to other types of malignancies have significant implications on long-term outcomes and allocation of health resources [2].

Small bowel NETs are a diagnostic problem because they can present in a non-specific manner in many cases. The patients can also be asymptomatic for a long period, and when they develop symptoms, they are often similar to more common gastrointestinal diseases like biliary disease and functional disorders [3]. Symptoms in secretory NETs can be associated with hormonal secretion diarrhoea, flushing, and vomiting. Non-secretory tumours, however, may be at clinical indolence and later become acute with obstruction of the bowel [4]. This diagnostic uncertainty usually leads to late diagnosis, and the diagnosis is often made late or by chance on the cross-sectional imaging [3].

The changing conceptualization of the NETs has given place to the emergence of standardized guidelines and multidisciplinary management approaches that mirror the increasing importance that is attached to them clinically [3]. However, the general medical and surgical teams have variable awareness, and the cases are still being diagnosed at late stages.

We present the case of a 49-year-old woman who came to the hospital with non-specific abdominal symptoms and was later diagnosed with cholecystitis because of the finding of gallstones in the ultrasound; she was later diagnosed with an ileal NET after emergency surgery was performed to fix the small bowel obstruction. The case demonstrates the diagnostic traps when incidental discoveries shift the clinical focus to the underlying pathology and the significance of having a wide-ranging diagnostic differentiation in patients with unexplained weight loss and chronic gastrointestinal symptoms.

## Case presentation

The patient, a 49-year-old female with no significant past medical or surgical history, presented to the Emergency department with a one-month history of progressive epigastric pain accompanied by nausea, vomiting, and reduced appetite. She complained of a significant unintentional weight loss of about 2 stones in the last one month. She was a non-smoker, did not take alcohol, and did not have any family history of any malignancy or metabolic disorders. She worked in the healthcare field and had not been admitted to the hospital before.

At first glance, the patient was haemodynamically stable. The examination of the abdomen found mild epigastric tenderness without guarding or peritonism. There were no palpable masses.

Prelude blood tests showed that liver tests were deranged, with alanine aminotransferase (ALT) of 79 IU/L and alkaline phosphatase (ALP) of 135 IU/L. Serum albumin was low at 23 g/L, and total protein was reduced to 47 g/L. Bilirubin was slightly high at 26 µmol/L. C-reactive protein had increased to 9.9 mg/L. Haematological work, such as the white blood cell count, haemoglobin, and platelet count, was normal. The full report of primary biochemistry and haematology is summarized in Tables 1, 2, respectively.

**Table 1:** Initial biochemistry results on admission Summary of biochemistry investigations performed on initial presentation to the emergency department. Patient values are presented alongside the corresponding normal reference ranges. ALP: alkaline phosphatase; ALT: alanine aminotransferase; CRP: C-reactive protein; e-GFR: estimated glomerular filtration rate; Free T3: free triiodothyronine; Free T4: free thyroxine.

Biochemistry	Patient Value	Normal Value
Albumin (g/L)	23	35-50
ALP (IU/L)	135	30-130
ALT (IU/L)	79	0-34
Amylase (IU/L)	14	10-90
Bilirubin (µmol/L)	26	0-20
Adjusted Calcium (mmol/L)	2.37	2.20-2.60
CRP (mg/L)	9.9	0.2-4.9
Creatinine (µmol/L)	56	49-90
e-GFR (mL/min/1.73 m^2^)	>90	>90
Free T3 (pmol/L)	2.9	3.5-6.5
Free T4 (mIU/L)	18.6	11.5-22.7
Globulin (g/L)	23	22-37
Phosphate (mmol/L)	1.10	0.8-1.5
Magnesium (mmol/L)	0.91	0.7-1.0
Potassium (mmol/L)	3.7	3.5-5.3
Folate (µg/L)	4.8	3-19
Sodium (mmo/L)	134	133-146
Total Protein (g/L)	47	60-80
Urea (mmol/L)	8.0	2.5-7.8

**Table 2:** Haematology results on admission Summary of haematological investigations performed on initial presentation. Patient values are presented alongside the corresponding normal reference ranges. WBC: white blood cell count; Hb: haemoglobin; PLT: platelet count; aPTT: activated partial thromboplastin time.

Haematology	Patient Value	Normal Value
WBC (×10^9^/L)	6.8	3.90-11.2
Hb (g/L)	126	120-156
PLT (×10^9^/L)	315	150-450
aPTT (seconds)	30.3	27-37

Further, an abdominal ultrasound was done, which showed the presence of gallstones and no biliary dilatation. Based on these findings, a provisional diagnosis of cholecystitis was made, and the patient was admitted under the medical team to investigate and manage the situation further. 

**Figure 1:**
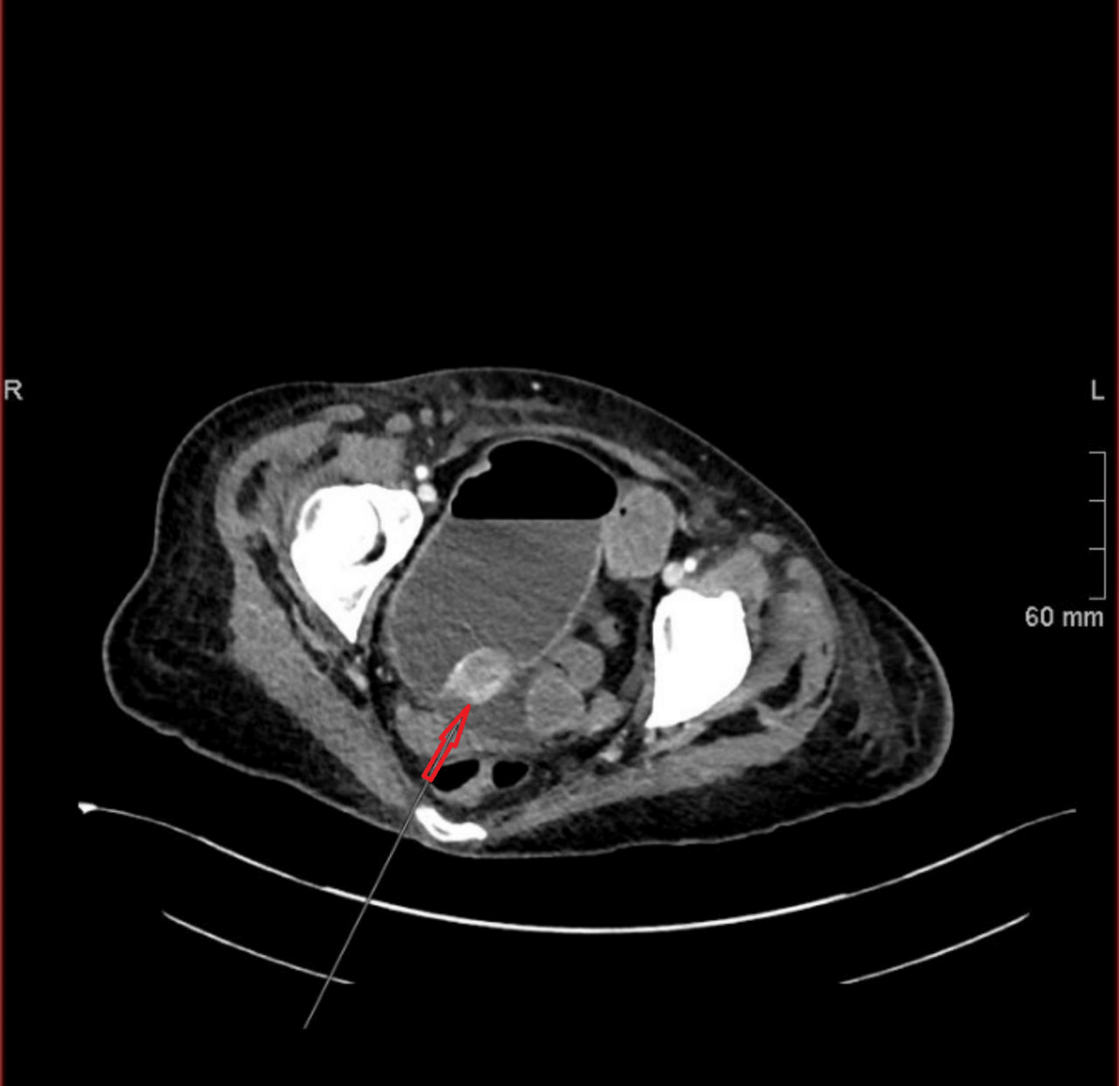
Computed tomography scan of the abdomen and pelvis demonstrating acute small bowel obstruction Axial computed tomography image of the abdomen and pelvis performed on the third day of admission following acute clinical deterioration. The image demonstrates dilated loops of small bowel with a transition point in the ileum (arrow), consistent with acute small bowel obstruction secondary to a stricturing ileal tumour. Mild ascites is also noted. CT: computed tomography.

The patient acutely deteriorated on the third day of admission with severe abdominal pain, progressive abdominal distension, intractable vomiting, and a significantly raised lactate level of 5.5 mmol/L. While the patient's initial biochemical picture and her later symptoms painted a picture of cholangitis/cholecystitis, her developing symptoms of intestinal obstruction warranted further cause for consideration, which magnetic resonance cholangiopancreatography (MRCP) alone would not have covered sufficiently. While MRCP was done following the initial chain of thought, i.e., cholecystitis complicating into possible gallstone ileus, computed tomography of the abdomen and pelvis (CTAP) was done to consider the unusual causes of obstructive picture, such as volvulus, ischemic colitis, and mesenteric ischemia. An abdominal and pelvis computed tomography (CT) scan was conducted urgently, and the appearance was that of acute small bowel obstruction (Figure 1). The MRCP showed the presence of a small number of calculi in the gallbladder and no biliary enlargement or common bile duct calculus. Bowel loops were observed to be enlarged in keeping with a mild level of ascites. Urgent surgical opinion was taken. 

The patient underwent emergency laparotomy. Intra-operative observation showed that there was a stricturing tumour of the ileum that covered the entire circumference of the bowel with lymphadenopathy in the respective mesentery. Surgical resection of the involved segment was done.

After the surgery, the patient was cared for in the high-dependency surgical unit. She started to develop acute confusion and behavioural changes, making her recovery difficult. An extensive workup was done to establish the underlying cause. Metabolic and biochemical screen testing, such as Chromogranin A, Chromogranin B, 5-hydroxyindoleacetic acid (5-HIAA), vasoactive intestinal peptide (VIP), somatostatin, noradrenaline, met-adrenaline, and glucagon, were within normal limits. Complete immunology panel that includes connective tissue disease screen, anti-glycolipid antibodies, anti-Ro-52 antibodies, liver-kidney microsomal (LKM) antibodies, soluble liver antigen, smooth muscle antibodies, mitochondria (M2) antibodies, and gastric parietal cell antibodies were all negative. The results of immunology and carcinoid markers are presented in Tables 3, 4, respectively.

**Table 3:** Immunology panel results Summary of immunological investigations performed during the post-operative diagnostic workup for acute confusion. All autoimmune markers were negative. LKM: liver-kidney microsomal; IgA: immunoglobulin A; M2: mitochondrial antibody type 2.

Immunology	Patient Value	Normal Value
Antibodies to Ro-52	Negative	<20cu
Anti-glycolipid antibodies	Negative	<1:500
Chromogranin A	18	0-59
Connective tissue disease screen	Negative	Negative
LKM antibodies	Negative	<1:10
Soluble liver antigen	Negative	0.0-20.0
Total serum IgA	3.03	0.8-4.0
Gastric parietal cell antibodies	Negative	Negative
Smooth muscle antibodies	Negative	Negative
Mitochondria (M2) antibodies	Negative	Negative

**Table 4:** Biochemical and carcinoid marker results Summary of neuroendocrine-specific biochemical markers measured during the post-operative period. All values were within normal limits, consistent with a non-functional neuroendocrine tumour. 5-HIAA: 5-hydroxyindoleacetic acid; VIP: vasoactive intestinal peptide.

Carcinoid Marker	Patient Value	Result
Chromogranin A	18	Normal (0-59)
Chromogranin B	Within normal limits	Normal
5-HIAA	Within normal limits	Normal
VIP	Within normal limits	Normal
Somatostatin	Within normal limits	Normal
Noradrenaline	Within normal limits	Normal
Met-adrenaline	Within normal limits	Normal
Glucagon	Within normal limits	Normal

Neurological examination, along with MRI of the brain and the entire spinal cord, was normal and ruled out any metastatic or autoimmune pathology as the underlying cause. Analysis of cerebrospinal fluid (CSF) showed normal fluid without evidence of infection and abnormal cellularity, and the virology test was negative (Table 5).

**Table 5:** Cerebrospinal fluid analysis results Summary of cerebrospinal fluid analysis performed as part of the neurological workup for post-operative acute confusion. The fluid was clear with no evidence of infection or abnormal cellularity, and the virology screen was negative. RBC: red blood cells; WBC: white blood cells; cmm: cells per cubic millimetre; CSF: cerebrospinal fluid.

Cerebrospinal Fluid Analysis	Result
Appearance	Clear fluid
RBC/cmm	<1
WBC/cmm	1
Culture	No growth
Virology screen	Negative

No functional or anatomic aetiology of the confusion was discovered, and the episode was treated as post-operative delirium. Her psychological situation then improved gradually with supportive measures, and the rest of her postoperative recovery was without any incidents.

The case was proven with histopathological analysis of the resected specimen, which confirmed the diagnosis of an ileal NET: well-differentiated NET, G1, pT3 pN1, R0.

Histopathological analysis of the resected specimen confirmed the diagnosis of an ileal NET. Sections examined using microscopy revealed ileal tissue that had a tumour in a predominantly nested growth pattern and which was characterized by bland monotonous cells with round nuclei and salt-and-pepper chromatin. The penetration of the tumour cells was deep into the muscularis propria, and focal invasion of the subserosa was also noted. A total of five lymph nodes were identified, three of which were positive for tumour cells. There was prominent lymphovascular space invasion. 

The morphological and immunohistochemical profile was consistent with a well-differentiated NET, Grade 1, with a Ki-67 proliferation index of less than 3%, in keeping with the lowest-risk category as defined by the WHO 2022 Classification of Digestive Tumours.

A thoracic-abdominal-pelvic CT scan that was conducted one month after surgery revealed no signs of residual disease or recurrence. The case was then presented in the NET multidisciplinary team (MDT) meeting, which suggested follow-up with a yearly surveillance CT scan, along with annual serum Chromogranin A and urinary 5-HIAA levels in accordance with international guidelines (ENETS 2024 and NANETS 2017).

## Discussion

NETs are a heterogeneous group of neoplasms arising out of neuroendocrine cells, with the gastrointestinal tract constituting the most common place of origin [1]. Even though the occurrence of NETs was historically regarded as rare, the number of such cases has been increasing significantly over the last several years. The evidence in England shows that the incidence of this disease, age-adjusted, grew 3.7 times in the 1995-2018 period, making a step up to 2.35 to 8.61 per 100,000 population [2]. This has been described by an increase in diagnostic imaging, an increase in awareness among clinicians, and an enhancement in histopathological classification. The survival rates are relatively high with NETs relative to other malignancies, which creates some significant concerns about follow-up and survivorship care, as well as the allocation of healthcare resources [2].

Small bowel NETs are a rather problematic condition to diagnose due to the often non-specific clinical manifestations of the condition. The symptoms can be vague and insidious and may include abdominal pain intermittently, weight loss, and bowel alterations, which are similar to other more common gastrointestinal disorders [3]. In secretory NETs, symptoms related to hormonal secretion, such as diarrhoea, flushing, and periodic vomiting, could be directly related to the clinical manifestation. Non-secretory tumours, however, as is the case with our patient, may still be asymptomatic over a long period before it becomes acute, with complications like small bowel obstruction [3]. The failure to develop carcinoid syndrome in our patient and the normal level of biochemical markers of Chromogranin A, 5-HIAA, VIP, and somatostatin are also in line with an inactive tumour, and also explain why such lesions go so easily missed during the early diagnostic investigation.

The first clinical course in our case is especially educative. Our patient had non-specific gastrointestinal symptoms, and after the discovery of the presence of gallstones during abdominal ultrasound, a provisional diagnosis of cholecystitis was made, which seemed to provide a reasonable answer to her symptoms. This brings to the fore a diagnostic fallacy of clinical practice in which the incidental findings are likely to be misleading and hence distract clinical practice focus from the actual underlying pathology [4]. Gallstones that are present in about 10-15% of the adult population may not necessarily give a causal relationship with the symptoms present in a patient, especially in cases where the patient has unexplained weight loss and hypoalbuminaemia as well. This case is an example of reminding clinicians that they need to be cautious in blaming symptoms on incidental findings, particularly in cases when the clinical picture does not align well with the diagnosis.

The diagnostic workup of the NETs is based on the synthesis of biochemical markers and radiological investigations. Chromogranin A is the most common general biomarker used to test NETs, whereas 5-HIAA is more useful in testing serotonin-secreting tumours [5]. But as it has been shown in our case, these markers can be fully normal in non-functional tumours and hence cannot be used as screening tools in the event of no clinical suspicion. Cross-sectional CT and MRI imaging are very important in tumour localization, staging, and surgical planning despite the fact that small bowel primary tumours may be exceptionally difficult to appreciate in primary imaging, especially before they develop obstructive complications [3]. The first ultrasound and the following MRCP failed to detect the ileal tumour in our patient, and the diagnosis was only confirmed after emergency surgery.

Surgery has been established as a component of the treatment of small bowel NETs. Localized and locoregional disease is still treated with surgical resection as the primary line of therapy, which has a chance of curing the disease and accessing tissue, which can be used to make a definitive histopathological diagnosis [6]. In our situation, the acute obstruction of the small bowel was the reason that led to the emergency laparotomy, and the intra-operative observation showed a stricturing tumour of the ileum with mesenteric lymphadenopathy. An excellent resection of the affected segment was realized, and the staging CT later revealed the absence of residual or metastatic disease. Formal oncological resection with lymph node sampling is currently recommended as the nodal involvement of the mesenteric area is common even in the case of early-stage disease [6]. The histopathologic confirmation of Grade 1 well-differentiated NET in our patient has a favourable prognostic value, but long-term follow-up is required due to the possibility of late recurrence.

Acute confusion developed in our patient after the surgical intervention, and a comprehensive diagnostic investigation was performed. Post-operative delirium is a comparatively frequent condition in the situation of a major abdominal surgery and can be triggered by a variety of issues such as metabolic imbalances, infection, drugs, and even the physiological stress of major surgeries [7]. In our patient, a detailed examination, including metabolic screening, immunological workup, CSF analysis, and MRI of the brain and whole spine, failed to show the underlying structural or metabolic aetiology. The episode had thus been explained by a case of post-operative delirium, which had cleared under the influence of supportive treatment. This detail of the case indicates that the systematic approach to altered mental status in the post-operative environment is crucial, as it is necessary to exclude the treatable causes of symptoms and diagnose symptoms as a diagnosis of exclusion.

MDT approach is the most acknowledged gold standard of managing NETs, and it helps in the coordinated input of surgical, oncological, radiological, and pathological specialists [4]. Our patient was discussed at a specific NET MDT meeting after the histopathological confirmation, and a systematic surveillance plan should be organized that includes regular-interval imaging. Such a collaborative methodology will guarantee that the decision-making process of the management is evidence-based, personalized, and aligned with the latest guidelines.

The Ki-67 proliferation index is the pivotal determinant of both prognosis and treatment selection in NET management. Per the WHO 2022 classification, Grade 1 tumours (Ki-67 less than 3%) have the most favourable long-term outcomes and, following curative resection, are managed with structured surveillance alone without the need for adjunctive systemic therapy [8]. Grade 2 tumours (Ki-67 3-20%) carry an intermediate prognosis; progressive or metastatic G2 disease warrants somatostatin analogue (SSA) therapy with octreotide or lanreotide, which have been demonstrated to significantly prolong progression-free survival in the landmark PROMID and CLARINET randomized controlled trials, respectively. Grade 3 well-differentiated NETs and poorly differentiated neuroendocrine carcinomas (NECs) require more aggressive systemic strategies, including platinum-based chemotherapy for NECs and, in SSTR-positive tumours, peptide receptor radionuclide therapy (PRRT) with 177Lu-DOTATATE, which demonstrated a significant progression-free survival benefit in the NETTER-1 phase III trial. It is, therefore, essential that Ki-67 assessment is formally documented on all NET histopathological reports, as it defines the entire clinical management trajectory. In the present case, confirmation of G1 disease provides prognostic reassurance and appropriately directs management towards surveillance without systemic therapy [9,10].

Regarding post-operative surveillance, ENETS 2024 and NANETS 2017 provide clear, evidence-based protocols for patients following curative resection of Si-NETs [9,10]. For Grade 1 tumours without residual disease on post-operative CT, the recommended surveillance programme includes annual serum Chromogranin A and urinary 5-HIAA to detect early biochemical recurrence; cross-sectional imaging with contrast-enhanced CT or MRI of the abdomen and pelvis at 12-24-monthly intervals for the first several years; and 68Ga-DOTATATE PET/CT somatostatin receptor imaging (SRI) when biochemical markers become abnormal or when cross-sectional imaging findings are equivocal. SRI has superseded OctreoScan (111In-pentetreotide scintigraphy) in all staging and surveillance indications per the joint NANETS/SNMMI Appropriate Use Criteria [11]. In the present case, a post-operative 68Ga-DOTATATE PET/CT is additionally recommended to complete formal staging, given that pre-operative functional imaging was not possible in the acute emergency setting. These structured surveillance protocols reflect the recognition that even G1 Si-NETs carry a risk of late recurrence, underscoring the importance of long-term, guideline-concordant follow-up [9,10].

Altogether, this case highlights the need to pay attention to the use of small bowel NETs in the differentiation of patients with unexplained abdominal symptoms and bowel obstruction, even in cases when there are apparently etiologic gallstones. Clinicians must exhibit a high index of suspicion, especially when dealing with patients whose symptoms are persistent or progressive when they are out of proportion to initial results. Increased prevalence of NETs requires increased attention from medical and surgical staff, and prompt referral to further imaging or an early surgical check-up should be taken into account in case the clinical image does not correspond to the initial working diagnosis.

## Conclusions

Small bowel NETs can be difficult to diagnose due to their non-specific presentation and the potential to remain clinically silent until complications develop. Incidental findings on imaging may contribute to diagnostic distraction, potentially delaying recognition of the underlying condition.

In patients presenting with persistent abdominal symptoms, unexplained weight loss, and laboratory abnormalities that are not fully explained by the initial working diagnosis, it is important to maintain a broad differential diagnosis. A systematic and comprehensive approach is essential when evaluating post-operative complications, including altered mental status, to ensure that reversible causes are considered before attributing symptoms to post-operative delirium.
